# Management of pediatric brain arteriovenous malformation: a systematic review of retrospective studies

**DOI:** 10.1007/s10143-026-04346-2

**Published:** 2026-05-28

**Authors:** Helbert de Oliveira Manduca Palmiero, Eberval Gadelha Figueiredo

**Affiliations:** Division of Neurosurgery, University of São Paulo Medical School, Dr. Enéas de Carvalho Aguiar Ave., 255, Room 5083, São Paulo, 05403-000, Brazil

**Keywords:** pediatric brain arteriovenous malformations (bAVMs), retrospective cohort studies, microsurgical resection, stereotactic radiosurgery (SRS), endovascular embolization, hemorrhagic presentation

## Abstract

**Introduction:**

Pediatric brain arteriovenous malformations (bAVMs) are a major cause of hemorrhagic stroke in children, yet management decisions rely largely on heterogeneous cohorts. Contemporary retrospective studies were systematically reviewed to summarize outcomes across treatment modalities and to characterize interstudy variability.

**Methods:**

PubMed/MEDLINE was searched over the past decade for retrospective pediatric intracranial bAVM cohorts. Eligible studies reported management with observation/conservative care, endovascular embolization, microsurgical resection, stereotactic radiosurgery (SRS), or multimodality therapy. Screened studies had data extracted using a standardized form. Outcomes included obliteration, hemorrhage (first presentation and post-treatment), functional outcomes, complications, and recurrence after apparent cure. Treatment categories were harmonized into mutually exclusive groups for descriptive synthesis.

**Results:**

Of 113 identified records, 20 retrospective cohort studies met the inclusion criteria. Hemorrhage was the most common presentation. Across cohorts reporting Spetzler–Martin (SM) grade, low-grade lesions (SM I–II) comprised 740 patients (44.1%), SM III 645 (38.4%), and SM IV–V 293 (17.5%). Treatment strategies varied substantially; among 1,973 classifiable patients, cohorts reported microsurgery-only, SRS-only, endovascular-only, multimodality, and conservative approaches. Definitions of obliteration and assessment methods were inconsistent (DSA-confirmed vs. MRI-based). Microsurgical series reported obliteration rates of 81%–100% in selected populations (unweighted mean ~ 93% across reporting cohorts), whereas SRS-only cohorts demonstrated obliteration rates of 51%–68.5% in the three largest unselected pediatric series (range 36%–84% across all SRS-inclusive cohorts), with latency-period hemorrhage rates of approximately 1.1%–3.2% per series. Recurrence after apparent cure was reported in 7 cohorts, with rates ranging from < 1% to 29%, occurring years after angiographic obliteration.

**Conclusions:**

Contemporary retrospective pediatric bAVM data indicate that modality-specific outcomes are strongly influenced by lesion selection, outcome definitions, and follow-up duration. Recurrence after apparent cure is not uncommon and supports explicit long-term surveillance. Standardized definitions and harmonized reporting are needed to improve interpretability and comparative inference.

## Introduction

Brain arteriovenous malformations (bAVMs) are high-flow congenital vascular lesions defined by a nidus of dysplastic vessels supplied by arteries and drained by veins, without an intervening capillary network [1–3]. Although uncommon in children (estimated prevalence 0.014%–0.028%), pediatric bAVMs account for a disproportionate initial presentation of hemorrhagic stroke, representing approximately half of intracranial hemorrhages in this population [3, 4]. Hemorrhage is clinically consequential: pediatric rupture has been associated with permanent neurological deficits in roughly 20%–41% and with mortality approaching 25% [3, 5]. Across reported pediatric cohorts, the annual hemorrhage risk varies widely (approximately 0.9%–7.14%), reflecting heterogeneity in angioarchitecture and patient-level risk factors [3]. Moreover, hemorrhagic presentation is not random—meta-analytic pediatric data indicate that rupture at presentation is associated with smaller nidus size, deep or infratentorial location, deep venous drainage, and diffuse morphology, among other features [5]. These natural-history considerations are amplified in children by their long life expectancy and the resulting cumulative exposure to hemorrhage over decades [4].

Contemporary management is inherently multimodal, employing microsurgical resection, endovascular embolization, and stereotactic radiosurgery (SRS), alone or in combination, guided by lesion size, eloquence, and complexity [3, 6]. Large multicenter radiosurgery experience supports SRS as an effective option—particularly for lesions not amenable to safe resection—with long-term obliteration observed in a majority of treated pediatric patients and a relatively low annual latency-period hemorrhage rate after SRS (≈ 1.1% per year) [4]. At the same time, institutional multimodality series report that embolization, SRS, and surgery are frequently used in a complementary fashion, with angiographic obliteration achievable in a substantial proportion of children [7]. A distinct pediatric concern is durability: recurrence after an apparent angiographic cure has been reported in approximately 10%–15% of children, reinforcing the need for long-term surveillance and careful interpretation of “cure,” particularly when treatment is staged or multimodal [6, 7].

Despite these principles, decision-making remains complex because the pediatric evidence base is dominated by single-center retrospective cohorts with heterogeneous populations, variable outcome definitions, and limited time-dependent analyses [4]. In addition, competing risks of hemorrhage, treatment-related morbidity, and late events, such as recurrence, complicate direct comparisons across modalities [3, 6]. Accordingly, we conducted a systematic review of retrospective studies on pediatric bAVM management to synthesize real-world outcomes across treatment strategies, examine methodological heterogeneity that limits interpretability, and clarify what the existing retrospective literature can (and cannot) support when counseling children and families about optimal management [4, 6].

## Methods

This systematic review was conducted and reported in accordance with the PRISMA 2020 statement [8].

### Literature search strategy

 A structured search of PubMed/MEDLINE was conducted to identify retrospective cohort studies on the management of bAVMs. The publication window was restricted to January 1, 2015, through the date of the search (planned through 2025) to reflect contemporary practice patterns. The complete PubMed/MEDLINE search strategy combined controlled vocabulary and keywords for *brain arteriovenous malformation*, *intracranial/cerebral location*, *pediatric populations*, and treatment modalities (*conservative management/observation*,* microsurgical resection*,* endovascular embolization*,* and stereotactic radiosurgery [SRS]*), along with filters for *retrospective studies* and *cohort* designs.

### Eligibility criteria

Studies were eligible if they: (1) reported on pediatric patients with intracranial bAVMs; (2) described management with observation/conservative care, endovascular embolization, microsurgical resection, SRS, or combined (multimodal) approaches; (3) used a retrospective cohort design; and (4) included follow-up sufficient to report at least one post-management outcome. No minimum follow-up duration was required; eligibility was met if any post-management outcome (e.g., obliteration status, hemorrhage, functional outcome, or recurrence) was reported, regardless of assessment timing. Case reports, narrative reviews, and meta-analyses were excluded.

### Study selection 

**Fig. 1:**
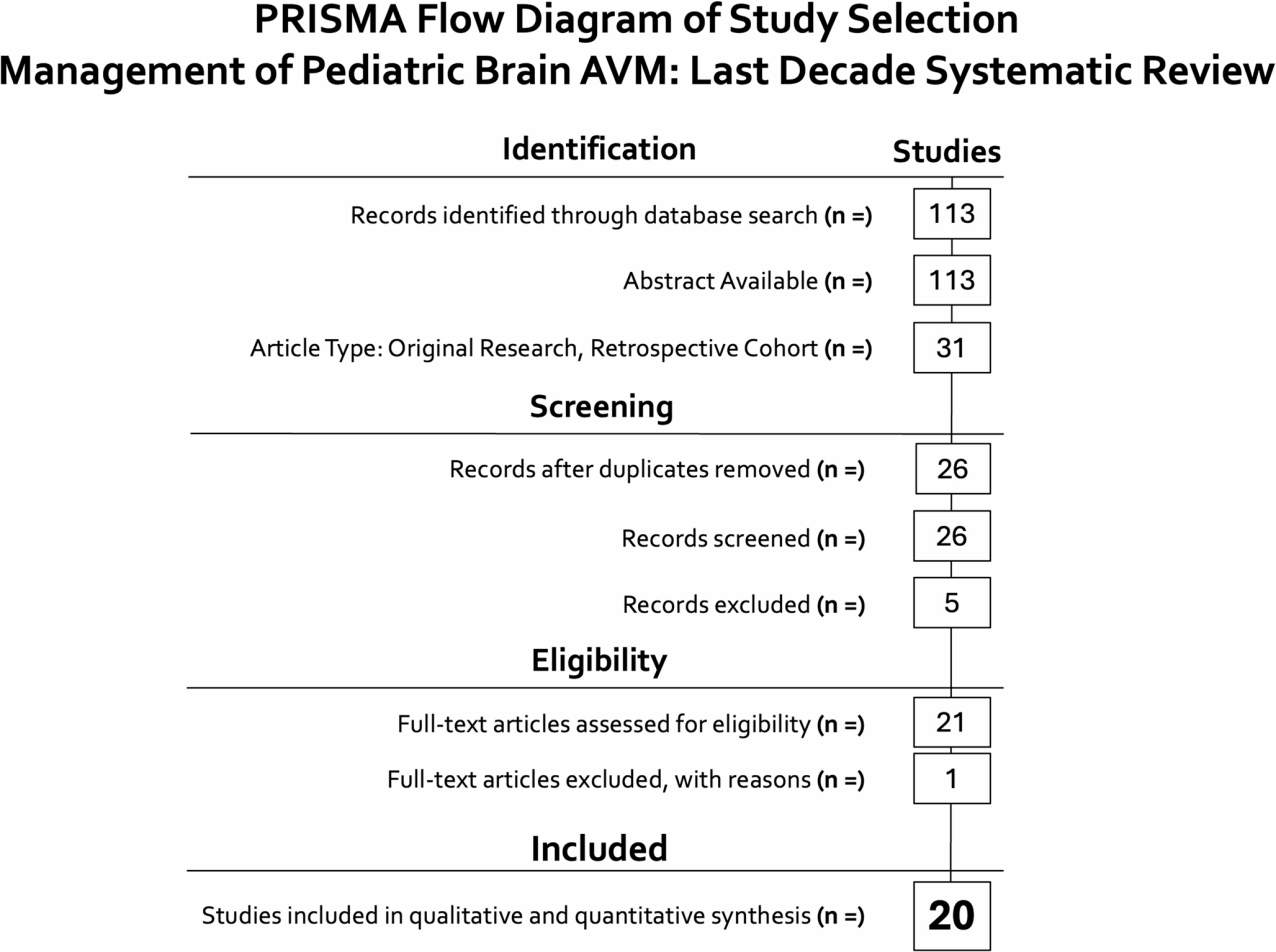
PRISMA 2020 flow diagram for study identification, screening, eligibility assessment, and inclusion in retrospective cohort studies on pediatric bAVM management (113 records identified; 20 studies included)

Two reviewers independently screened titles and abstracts, followed by full-text assessment against predefined eligibility criteria. Disagreements were resolved by consensus. Study selection was summarized in a PRISMA flow diagram **(**Fig. 1**)**.

### Data extraction and variables 

Data were extracted using a standardized form. Variables included study design and sample size; patient age, rupture status, presenting symptoms, and follow-up duration; and angioarchitectural characteristics (Spetzler–Martin grade, nidus size, deep venous drainage, eloquence, and deep location). Management variables comprised treatment modality (observation, embolization, microsurgery, SRS, or multimodal therapy), definitions of obliteration, clinical outcomes, and complications. For radiosurgery cohorts, technical parameters (platform and margin dose) were recorded when available. Studies reporting recurrence were summarized separately, including treatment received, hemorrhagic presentation, recurrence rate, and follow-up interval.

### Outcomes and synthesis

The primary analysis was descriptive. Outcomes included angiographic or radiographic obliteration, post-treatment hemorrhage (including latency-period hemorrhage after SRS), functional outcome measures, treatment-related complications, mortality, and recurrence after apparent cure. Treatment strategies were categorized into mutually exclusive groups based on single- or combined-modality therapy. Patients who underwent prior embolization before SRS were classified as receiving combined therapy to avoid double-counting.

## Results

The search identified 113 records; after screening and full-text review, 20 retrospective cohort studies met the inclusion criteria **(**Fig. 1**)**.

### Cohort Characteristics 

The included studies comprised single-center institutional series, multicenter registries, and international radiosurgery collaborations. Cohort sizes varied widely, ranging from small institutional experiences to large multi-institutional radiosurgery datasets. Study focus was heterogeneous and included microsurgery-dominant series, radiosurgery-only cohorts, endovascular-focused reports, multimodality experiences, conservative-management cohorts, and recurrence-focused analyses **(**Table 1**)**.

**Table 1:** Summary of retrospective cohort studies published over the past decade on the management of pediatric brain arteriovenous malformations (bAVMs)

Study	Retrospective Cohort (Setting)	*N*	Age (Years)	Follow-up	Ruptured	Symptoms, 1st	Hemorrhage
Ding (USA, 2016) [9]	Single-center Gamma Knife radiosurgery for partially resected AVMs − 1:1 matched to unresected AVMs	176	26.8 ± 11.7 (resected); 27.1 ± 13.5 (unresected)	Radio 77.7 ± 54.3 months (resec) vs. 80.6 ± 52.8 (unresec); Clinical 88.6 ± 58.0 vs. 92.8 ± 55.8	160/176 (90.9%)	Hemorrhage 87.5%, Headache 5.7%, Seizure 3.4%, Focal deficit 3.4%	77 (87.5%)
Patibandla (USA, 2017) [10]	Single-center single-session Gamma Knife SRS for pediatric SM grade IV AVMs	28	12.1 ± 3.7	115.5 months ± 51.5	18 (64.3%)	Hemorrhage 64.3%, Seizure 14.3%, Focal deficit 10.7%, Headache 3.6%, Asymptomatic 7.1%	18 (64.3%)
Starke (International, 2017) [11]	Multicenter Gamma Knife radiosurgery for pediatric AVMs (< 18y); >=12-month follow-up (IGKRF cohort)	357	12.6 ± 3.7 (2.8–17.9)	92.4 months ± 62.0, 12–266	245 (68.6%)	Hemorrhage 68.6%, Focal deficit 13.2%, Seizure 8.1%, Headache 5.3%, Asymptomatic 2.2%	245 (68.6%)
Oulasvirta (Finland, 2018) [12]	Population-based pediatric AVMs (< 18y at admission) - comparison with adult cohort (Helsinki cohort)	127	12.1 (2.3 months–18 years)	21.2 years (0–62.3 years)	97 (76%)	Hemorrhage 76%, Epilepsy 20%	97 (76%)
Umansky (Israel, 2018) [13]	Partially Onyx-embolized pediatric AVMs - subsequent stereotatic radiosurgery (combined modality cohort)	12 (14 total)	15.3 (8.4–20) at start of combined treatment	49.9 months (12.8–118.8); median 51 months	11/14 (78.6%)	Hemorrhage 78.6%, Seizures 14%, Focal deficit 7%	11/14 (78.6%)
Al-Smadi (USA, 2019) [14]	Single-center pediatric low-grade (SM I-II) bAVMs - combined preoperative embolization + microsurgical resection vs. surgery alone	34	10.6 ± 3.4 (3–16)	35.7 months (3–97)	20 (59%)	Headache 56%, Seizure 12%, Focal deficit 6%, ↓Consciousness 18%, Incidental 18%	20 (59%)
Deng (China, 2019) [15]	Single-center pediatric hemorrhagic bAVMs - microsurgical resection cohort (long-term outcomes)	111	11.1 ± 4.0 (1–18)	4.3 ± 2.1 years (1.2–8.7)	111 (100%)	Hemorrhage 100%	111 (100%)
Lopes (USA, 2015) [16]	Previously untreated cerebral AVMs - primary Onyx embolization with curative intent (AVMES development cohort)	39	41 (range 1–71)	6-month DSA for cure assessment	9 (23%)	Seizures 31%, Headache 21%, Focal deficit (Visual 15%, Weak/numb 13%, Speech 3%), Incidental 13%	9 (23%)
Copelan (USA, 2020) [17]	Patients < 25y with microsurgically resected, DSA-confirmed cured AVMs - recurrence study	115	14.9 (range 3.1–24.8)	2.3 years (0–15)	72 (63%)	Headache 71%, Seizures 22%	72 (63%)
Antkowiak (Poland, 2021) [18]	Single-center pediatric ruptured bAVMs - multimodal treatment (microsurgery, embolization, radiosurgery)	22	11.9 (range 2–17)	3 months (imaging post-treatment)	22 (100%)	Headache 72.7%, Focal deficit 36.4%, ↓Consciousness 27.3%, Seizures 13.6%	22 (100%)
Burke (International, 2021) [19]	Single-session Gamma Knife SRS for pediatric AVMs - SRS-only vs. prior embolization + SRS (multicenter IRRF cohort)	539	12.9 (SRS-only) vs. 12.5 (E + SRS)	86 months (SRS-only) vs. 85 months (E + SRS)	386 (71.6%)	NR (Hemorrhage predominant)	386 (71.6%)
LoPresti (USA, 2020) [20]	High-grade (SM IV-V) pediatric bAVMs - conservative management (natural history cohort)	14	11.14 (range 6–17)	32.17 months (range 9.43–79.10)	4 (28.6%)	Headache 50%, Focal deficit (Weak 35.7%, Visual 14.3%), Incidental 28.6%	4 (28.6%)
Arkar (Slovenia, 2022) [21]	Population-based pediatric AVMs (brain and spinal), multimodality management	12	9.1 (range 1 month–16.3 years)	3.5 years (range 0–11.5)	9 (75%)	Hemorrhage 58%, Focal deficit 17%, Seizure 8%, Headache 8%, Incidental 8%	7 (58%)
Lim (Singapore, 2022) [22]	Multimodality management with surgery-dominant approach	58	8.7 ± 4.2	7.7 years (median 5.7; IQR 3.9–10.1)	51 (87.9%)	Headache 60.3%, Seizure 32.8%, ↓Consciousness 24.1%, Focal deficit 20.7%, Incidental 10.3%	51 (87.9%)
Järvelin (Finland, 2023) [23]	Microsurgically resected bAVMs with angiographic cure - recurrence study	135	37 (0–70); pediatric (≤ 18y) *n* = 17 (12.6%)	1.27y (0.02–30.4) clinical; median record FU 18.56y (0.02–39.33)	79 (58.5%)	Hemorrhage 58.5%, Focal deficit 36.5% (Weak 18.8%)	79 (58.5%)
Oulasvirta (Finland, 2023) [24]	Pediatric/young adult AVMs (< 21) with DSA-verified cure - long-term recurrence (Helsinki cohort)	41	14.6 (IQR 12–19; range 7–21)	19.1 years (8.6–35.7)	29 (70.7%)	Seizure 29.3%	29 (70.7%)
Rodriguez-Calienes (Peru, 2023) [25]	Single-center pediatric low-grade (SM I-II) bAVMs - first-line stand-alone endovascular embolization (curative intent)	68	14 (≤ 18)	14 months (0.6–77)	51 (75%)	Headache 61.8%	51 (75%)
Flores-Milan (2024) [26]	Single-center pediatric AVM (< 18); ruptured vs. unruptured; timing of multimodal therapy (resection +/- embolization, SRS +/- embolization)	27	11.9 ± 3.7, 5–17	41 months (IQR 20.3–79) ruptured; 47.5 months (IQR 19.8–55.8) unruptured	21 (77.8%)	Ruptured: Focal deficit 66.7%; Unruptured: Seizures 50%, Headache 33%, Incidental 16.7%	21 (77.8%)
Garcia (Internationtal, 2024) [27]	Pediatric residual AVM after prior SRS - repeat Gamma Knife radiosurgery	83	3–17 (at first SRS), 12–17 (at repeat SRS)	57 months (after SRS), 6-217	42 (50.6%)	Hemorrhage 49.4%, Headache 34.9%, Focal deficit 27.7% (Weak), Seizures 14.5%	41 (49.4%)
Kim (USA, 2025) [28]	Single-institution pediatric AVM treated with robotic (CyberKnife) radiosurgery	95	13.4 ± 4.2, 0–18	54 months, 21.6-182.4	44 (46.3%)	Headache 60%, Focal deficit 52.6%	44 (46.3%)

Studies are grouped by treatment strategy and study design. Age and follow-up are reported as mean ± SD or median (range/IQR), as reported in the original manuscripts. “Ruptured” denotes the proportion of patients presenting with hemorrhage. “Symptoms, 1st” indicates the presenting clinical manifestations at diagnosis. Hemorrhage column reports the number and percentage of patients with hemorrhagic presentation. Abbreviations: E embolization, FU follow-up, GKRS Gamma Knife radiosurgery, IGKRF International Gamma Knife Research Foundation, IQR interquartile range, IRRF International Radiosurgery Research Foundation, NR not reported, SRS stereotactic radiosurgery, SM Spetzler–Martin grading system

In studies reporting presentation, hemorrhage was the most common initial manifestation. Rupture rates ranged from 23% (embolization-only series, Lopes [16]) to 100% (hemorrhage-focused microsurgical series, Deng [15]; multimodality ruptured cohort, Antkowiak [18]), with most cohorts reporting hemorrhagic presentation in 59%–88% of patients. Surgery-dominant and recurrence-focused microsurgical series were composed predominantly of ruptured lesions (59%–100%), whereas radiosurgery and multimodality cohorts reported a broader range (47%–79%). Follow-up duration ranged from 3 months (Antkowiak [18]) to a median of 19.1 years (Oulasvirta 2023 [24]), with recurrence-focused cohorts reporting surveillance extending beyond a decade (Oulasvirta 2023 [24]: median 19.1 years; Järvelin [23]: record follow-up up to 39 years).

### Angioarchitectural Characteristics 

**Fig. 2:**
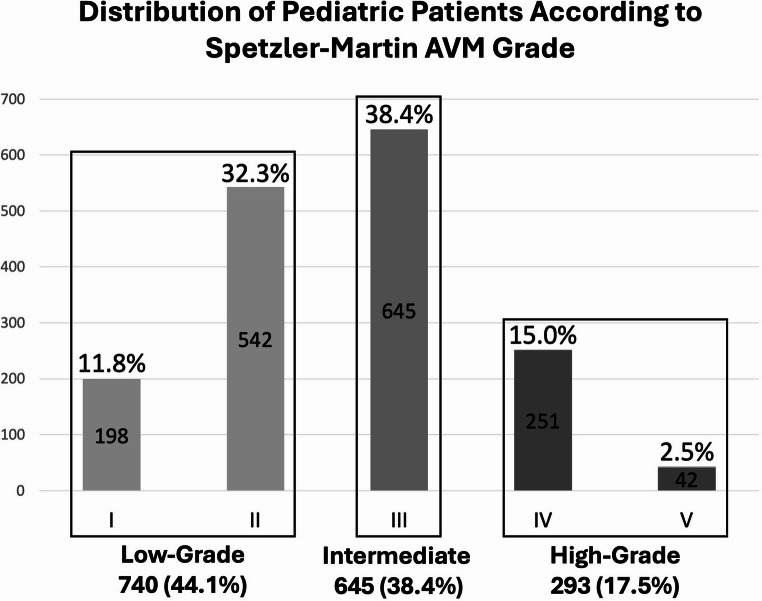
Distribution of pediatric patients according to Spetzler–Martin grade across the included retrospective cohorts. Low-grade lesions (Grades I–II) comprised 740 patients (44.1%), intermediate-grade lesions (Grade III) 645 patients (38.4%), and high-grade lesions (Grades IV–V) 293 patients (17.5%). Individual grade counts and percentages are shown for each category

Spetzler–Martin (SM) grade distribution was reported in most studies **(**Table 2**)**. Pooled grade counts across cohorts demonstrated that low-grade lesions (SM I–II) comprised 740 patients (44.1%), intermediate-grade lesions (SM III) 645 patients (38.4%), and high-grade lesions (SM IV–V) 293 patients (17.5%) **(**Fig. 2**)**. Low-grade lesions predominated in microsurgical and select endovascular cohorts, consistent with established treatment selection paradigms. Radiosurgery series included a broader distribution of SM grades, with substantial representation of SM III lesions and a nontrivial proportion of higher-grade malformations. High-grade lesions were overrepresented in conservative-management cohorts.

**Table 2:** Angioarchitectural profile of treated brain arteriovenous malformations (bAVMs) across included studies

Study	Grade (Spetzler-Martin)	Nidus Volume, Diameter (Mean ± SD)	Deep venous drainage (%)	Eloquent area (%)	Deep location (%)
Ding [9]	Low 87 (49.5%), Int 76 (43.2%), High 13 (7.4%)	V 2.6 ± 2.0 cm3 (Resect); 2.6 ± 3.3 cm3 (Unresect); D 2.0 ± 0.8 cm	112 (63.6%); 62.5% (Resect) vs. 64.8% (Unresect)	117 (66.5%); 68.2% (Resect) vs. 64.8% (Unresect)	53 (30.1%); 17 (19.3%, Resect), 36 (40.9%, Unresect)
Patibandla [10]	Low 0 (0%), Int 0 (0%), High 28 (100%)	V 5.9 ± 4.4 cm3; D 3.8 ± 0.8 cm	28 (100%)	28 (100%)	19 (67.9%)
Starke [11]	Low 147 (41.1%), Int 167 (46.8%), High 43 (12.1%)	V 3.5 ± 3.3 cm3; D 2.3 ± 1.5 cm	234 (65.5%)	276 (77.3%)	97 (27.2%, Thalamus/BG); Brainstem 36 (10.1%)
Oulasvirta [12]	Low 39 (34%), Int 44 (37%), High 35 (29.6%)	NR; Small 39%; Medium 44%; Large 17%	38 (31%)	NR	38 (31%)
Umansky [13]	Low 3 (25%), Int 2 (16.7%), High 7 (58.3%)	V SRS target 0.6 cm3 (0.1–7.3); V reduction after Onyx 66.7%	NR	NR	NR (BG and IV included)
Al-Smadi [14]	Low 34 (100%), Int 0 (0%), High 0 (0%)	NR; <3 cm 79%; 3–6 cm 21%	4 (12%)	12 (35%)	NR [Infratentorial 2 (6%)]
Deng [15]	Low 72 (64.9%), Int 25 (22.5%), High 14 (12.6%)	D 3.4 ± 1.4 cm	28 (25.2%)	41 (36.9%)	NR [Infratentorial 10 (9%)]
Lopes [16]	NR	D Scored: <3 cm, 3–6 cm, >6 cm (AVMES variable)	NR	NR	NR
Copelan [17]	NR	D 2.59 cm (0.4–10 cm)	19/85 (22%)	43/82 (52%)	NR
Antkowiak [18]	Low 15 (68.3%), Int 3 (13.6%), High 4 (18.2%)	D 0–3 cm 91%; 3–6 cm 9.1%; >6 cm 0%	9 (41%)	7 (31.8%)	4 (18.2%)
Burke [19]	Low 213 (39.5%), Int 243 (45.1%), High 83 (15.4%)	V 5.3 cm3 (SRS-only) vs. 8.4 cm3 (E + SRS)	345/539 (64%) overall; SRS-only 65.4%, E + SRS 57.1%	409/539 (75.9%) overall	182/535 (33.8%)
LoPresti [20]	Low 0 (0%), Int 0 (0%), High 14 (100%)	D 4.9 cm; 71.4% 3–6 cm; 28.6% >6 cm	14 (100%)	13 (92.9%)	9 (64.3%)
Arkar [21]	Low 3 (37.5%), Int 2 (25%), High 3 (37.5%)	D range 0.9–6.0 cm	NR	NR	3 (25%)
Lim [22]	Low 40 (68.9%), Int 13 (22.4%), High 5 (8.6%)	D <3 cm 79.3%; 3–6 cm 17.2%; >6 cm 3.4%	35 (60.3%)	27 (46.6%)	11 (19%)
Järvelin [23]	NR	NR	NR overall (50% among recurrences)	NR	NR
Oulasvirta [24]	Low 21 (51.2%), Int 15 (36.6%), High 4 (9.8%)	D 2.9 cm (IQR 19.5–40.3; range 0.8–6.5 cm)	15 (36.6%)	NR	7 (17.1%)
Rodriguez-Calienes [25]	Low 68 (100%), Int 0 (0%), High 0 (0%)	D 2.1 cm (IQR 1.5–2.7)	12 (18.2%)	21 (32.3%)	4 (5.9%)
Flores-Milan [26]	Low 17 (62.9%), Int 6 (22.2%), High 4 (14.8%)	D: Rupt 2.1 cm (IQR 1.5–2.8); Unrupt 2.9 cm (IQR 2.2–3.5)	13/27 (48.1%); Rupt 52.4%; Unrupt 33%	17/27 (63%); Rupt 57.7%; Unrupt 83%	5/27 (18.5%); Rupt 85.7%; Unrupt 100%
Garcia [27]	Low 36 (43.3%), Int 40 (48.2%), High 7 (8.4%)	V 4.5 cm3 (first SRS), 1.6 cm3 (repeat SRS)	NR	NR	37 (44.6%)
Kim [28]	Low 17 (18.1%), Int 34 (36.2%), High 43 (45.7%)	V 10.0 ± 11.88 cm3 (range 0.11–71.86)	NR		

Spetzler–Martin (SM) grade is categorized as Low (grades I–II), Intermediate (grade III), and High (grades IV–V). Nidus size is reported as mean ± SD or median (range/IQR), as provided in each study. Deep venous drainage, eloquent location, and deep location are reported as numbers (percentages) when available. Deep locations include the basal ganglia (*BG*), thalamus, and brainstem, unless otherwise specified by the original study. *BG* basal ganglia, *D* Diameter, *IQR* interquartile range, *NR* not reported, *SRS* stereotactic radiosurgery, *V* Volume

Reporting of angioarchitectural features beyond SM grade—including nidus size, deep venous drainage, eloquent location, and deep location—was inconsistent. Nidus size was reported as either maximum diameter or calculated volume, with inconsistent categorization thresholds. Deep venous drainage and eloquent location were more often reported in radiosurgery-heavy and high-grade cohorts. This heterogeneity precluded quantitative pooling of individual angioarchitectural risk factors.

### Treatment Distribution 

**Fig. 3:**
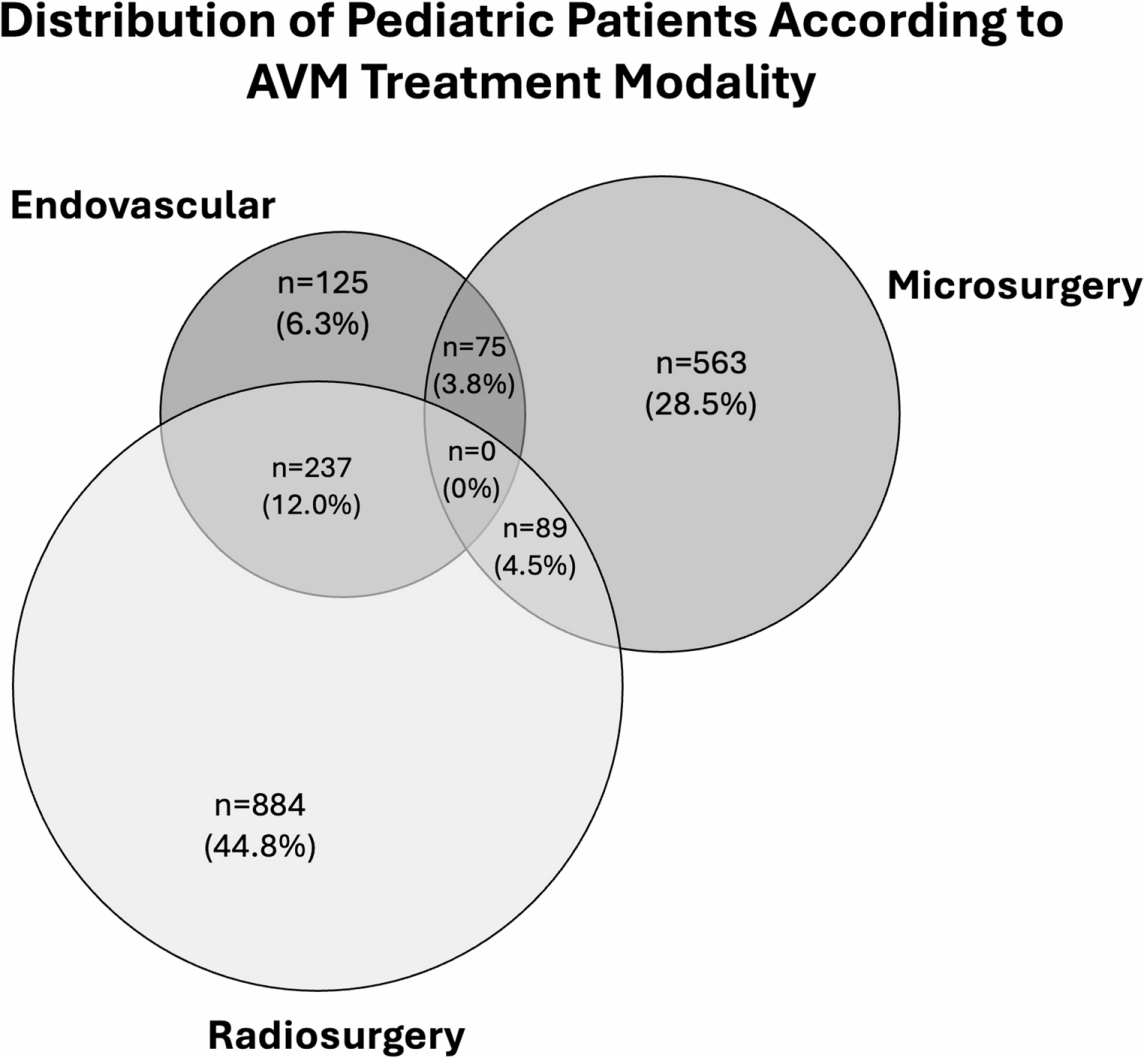
Distribution of pediatric patients according to AVM treatment modality. A venn diagram illustrating mutually exclusive treatment categories. Radiosurgery-only cases were adjusted by subtracting patients with prior endovascular therapy to avoid double-counting. Percentages are calculated relative to the total classifiable cohort (n = 1,973). No study reported triple-modality treatment (Endovascular+Microsurgery+Radiosurgery)

Treatment modality distribution varied substantially across cohorts **(**Table 3**)**. Among the subset of studies that could be categorized into mutually exclusive treatment groups for cross-cohort visualization, 1,973 patients were classifiable **(**Fig. 3**)**.

**Table 3:** Summary of treatment strategies and outcomes for pediatric brain arteriovenous malformations (bAVMs)

Study	Conservative (%)	Endovascular (%)	Microsurgery (%)	Radiosurgery (%)	Combined (%)	Notes (Obliteration, Outcomes)
Ding [9]	0	0	88 (50%) alone	88 (50%) alone; 88 micro+radio (50%)	All resec prior Micro, No prior Endo included, Repeat SRS 13.6% (resec) vs. 8.0%	84% Obliteration (resected); 1.1% annual hemorrhage; prior resection protected RIC; smaller size predicted obliteration; no cohort difference
Patibandla [10]	0	0	0	28 (100%)	Prior-Endo 10.7%, Prior-Micro 14.3%, Prior-EBRT 10.7%, repeat SRS 35.7%	36% Obliteration; age predicted success; 3.2% hemorrhage; 3.5% morbidity/mortality; ruptured higher obliteration
Starke [11]	0	0	0	357 (100%)	Prior-Endo 21.8%, Prior-Micro 6.4%, Prior-EBRT 13.2%	63% Obliteration; 1.4% hemorrhage; 0.8% mortality; ≥22 Gy improved outcomes; no embolization favored success
Oulasvirta [12]	0	4 (31%)	67 (52.8%)	2 (16%)	Micro ± Endo ± Radio, Conserv 25 (20%)	Pediatric: 47% long-term mRS < 2; 21% mortality; small AVM, surgery favorable; conservative care higher mortality
Umansky [13]	0	12 (85.7%)	0	14 (100%)	85.7% Endo Onyx + SRS	71% Obliteration; transient edema only; mRS improved 2→0; no hemorrhage or mortality
Al-Smadi [14]	0	0	34 (100%)	0	Endo + Micro 73.5%, Micro only 26.5%	30% complications; 6% permanent disability; no mortality; SM II and eloquence predicted complications
Deng [15]	0	0	111 (100%)	0	Micro only	100% Obliteration; Pediatric rupture risk 5.5–9.8%; 0.9% recurrence; 7% disability; early surgery improved short-term outcomes
Lopes [16]	0	39 (100%)	0	0	Endo only (Onyx)	67% Obliteration; 13% major complications; AVMES predicted outcomes (AUC 0.824); 84% good outcome
Copelan [17]	0	0	115 (100%)	0	Micro ±prior-Endo/SRS	10% recurrence; higher in young ruptured; risk decreased with age (HR 0.86)
Antkowiak [18]	0	4 (18.2%)	9 (40.9%)	3 (13.6%)	Micro 9, Endo 4, Radio 3, Endo+Micro 2, Endo + SRS 3, Micro + SRS 1	83.3% Overall Obliteration; 86% favorable outcome; no rebleeding, complications, or mortality; complete surgical obliteration on DSA
Burke [19]	0	91 (16.9%)	0	539 (100%): SRS-only *n* = 448, E + SRS *n* = 91	Prior-Endo 16.9% (91/539)	64.3% Obliteration (overall); SRS-only (307/448, 68.5%), SRS + E (39/90, 43.3%); embolization reduced cumulative obliteration
LoPresti [20]	14 (100%)	0	0	0	Conservative only	7% delayed hemorrhage/hydrocephalus; 14% seizures; 86% independent; no definitive AVM treatment performed
Arkar [21]	2 (17%)	6 (50%)	4 (33.3%)	0	Endo 50%, Micro 33%, Conserv 17%	25% Obliteration; none with embolization alone; residual deficits common; one death
Lim [22]	0	2 (34%)	36 (62.1%)	10 (17.2%)	Micro 62.1%, SRS 17.2%, Endo 3.4%, Surveillance 17.2%	81% Obliteration; 13% recurrence; 3% rebleeding; 86% favorable outcome; two deaths
Järvelin [23]	0	0	135 (100%)	0	Preoperative Endo 35.3%	4% recurrence (29% pediatric); mean 7.5 years; younger age and hemorrhage predicted recurrence; all re-occluded
Oulasvirta [24]	0	2 (4.9%)	37 (90.2%)	1 (2.4%)	14 (34.1%); mostly Micro, 2 Endo-only, 1 Radio-only	Late recurrence 5–7%; all initially ruptured, microsurgical; detected 14–26 years; minimal DSA complications
Rodriguez-Calienes [25]	0	68 (100%)	0	0	Endo only	65% Obliteration; 6% hemorrhagic complications; no mortality; smaller size, single vein favored success
Flores-Milan [26]	0	14 (51.9%): Rupt (10), Unrupt (4)	23 (85.2%): Rupt (18), Unrupt (5)	4 (14.8%): Rupt (3), Unrupt (1)	Pre Endo 47.6% (ruptured group)	25/27 92.6% Obliteration Overall; Ruptured: 95% good outcome, 5% mortality, 9% recurrence; Unruptured: 100% good, no recurrence/mortality
Garcia [27]	0	0	0	83 (100%)	Endo 18.1%, Micro partial 7.2%, CSF diversion 3.6%	51% Obliteration; 45% favorable; 12% latency hemorrhage; 30% RIC; 7% cyst; 18% retreatment; one death
Kim [28]	0	0	0	95 (100%)	Endo 49.5%, Micro 22.1%	53% Obliteration; 32% ARE; hemorrhage highest in VMS; 1% mortality

Treatment categories are reported as the proportion of patients receiving conservative management, endovascular embolization, microsurgical resection, stereotactic radiosurgery (SRS), or combined/multimodal approaches, as described in each study. “Combined” includes sequential or adjunctive therapy (e.g., embolization plus microsurgery or SRS). Obliteration is defined as angiographic or imaging-confirmed AVM cure, as specified by individual studies. Clinical outcomes are summarized as reported (e.g., favorable outcome, modified Rankin Scale [mRS], recurrence, hemorrhage, radiation-induced changes [RIC], adverse radiation effects [ARE], mortality). Abbreviations: EBRT external beam radiation therapy; E embolization; FU follow-up; GKRS Gamma Knife radiosurgery; HR hazard ratio; mRS modified Rankin Scale; NR not reported; RIC radiation-induced changes; SM Spetzler–Martin; VMS Virginia Radiosurgery AVM Scale

Microsurgery-only cohorts largely comprised low- to intermediate-grade lesions, often presenting with hemorrhage. Radiosurgery-only series represented a substantial proportion of the pooled population and were predominantly composed of SM II–III lesions, with selected higher-grade cases. Endovascular stand-alone cohorts were smaller and typically involved carefully selected low-grade lesions. Multimodal approaches—most commonly embolization combined with microsurgery or radiosurgery—were reported in several institutional series. In all identifiable combined embolization-plus-microsurgery cohorts, embolization was performed with a preoperative adjunctive intent (to reduce intraoperative blood loss or facilitate surgical planning) rather than as a primary curative strategy followed by salvage resection; however, retrospective reporting does not consistently distinguish between planned preoperative sequencing and unplanned rescue surgery, and this distinction cannot be definitively established from available data. A conservative cohort consisted exclusively of high-grade lesions deemed unsuitable for definitive intervention.

### Obliteration outcomes

Definitions of obliteration varied across studies, ranging from angiographic cure, as confirmed by digital subtraction angiography (DSA), to radiographic absence of flow on MRI. Assessment timing also differed, particularly in radiosurgery cohorts, where latency to obliteration is expected. Microsurgical series reported obliteration rates of 81%–100% across reporting cohorts (unweighted mean approximately 93%); five of six reporting series exceeded 90%, including one hemorrhage-focused cohort reporting 100% angiographic obliteration (Deng [15]) and one multimodality cohort reporting 92.6% overall (Flores-Milan [26]). Endovascular stand-alone cohorts reported obliteration rates of 65%–67% in selected low-grade lesions (Rodriguez-Calienes 65% [25]; Lopes 67% [16]), with treatment-related complications documented in some series. Radiosurgery-only cohorts demonstrated a wider range: 36% (Patibandla [10], all SM IV; highly selected high-grade cohort), 51% (Garcia [27], repeat SRS), 53% (Kim [28], robotic SRS), 63% (Starke [11], IGKRF multicenter), and 68.5% (Burke [19], SRS-only subgroup); the unweighted mean across the three largest unselected pediatric SRS series (Starke [11], Burke-SRS-only [19], Garcia [27]) was approximately 61%. SRS following prior embolization was associated with lower obliteration: 43.3% versus 68.5% for SRS-only in the largest comparative dataset (Burke [19]). Repeat radiosurgery was reported in select cohorts (Garcia [27]; Kim [28]). Combined multimodal series reported 83.3% (Antkowiak [18]) and 92.6% overall (Flores-Milan [26]). Given heterogeneity in outcome definitions, imaging modalities, and follow-up duration across cohorts, direct cross-modality comparison of these rates is not appropriate; the values above are descriptive summaries within modality-specific practice contexts.

### Hemorrhage during follow-up

Post-treatment hemorrhage was reported in most studies **(**Table 3**)**. In microsurgical cohorts, rebleeding after confirmed angiographic cure was uncommon yet not absent, particularly during long-term follow-up. In the radiosurgery series, latency-period hemorrhage occurring before obliteration was consistently reported. Hemorrhage rates during the latency period ranged from approximately 1% to 3% across several cohorts, with higher rates observed in selected high-risk or retreatment populations. Mortality was infrequent but reported in isolated cases. The conservative high-grade cohort experienced delayed hemorrhagic events and hydrocephalus during follow-up, underscoring the cumulative hemorrhage risk in untreated lesions.

### Functional outcomes and complications 

Functional outcomes were reported variably using the modified Rankin Scale (mRS), the Glasgow Outcome Scale, or institutional definitions of a favorable outcome. Surgery-dominant cohorts generally reported favorable functional outcomes in most low-grade lesions, although neurological morbidity was not negligible. Treatment-related complications varied by modality. Microsurgical complications included new neurological deficits and perioperative morbidity. Endovascular cohorts reported procedure-related hemorrhage and ischemic events. Radiosurgery cohorts documented adverse radiation effects (AREs), including radiographic changes with or without clinical symptoms. Symptomatic radiation-related changes were uncommon but present. Rare late cyst formation was described in select long-term radiosurgery series.

### Radiosurgery technical parameters 

Among radiosurgery studies **(**Table 4**)**, the Gamma Knife was the most commonly reported platform, although linear accelerator–based systems and robotic radiosurgery were also represented. Margin doses generally ranged from the high teens to the low twenties (Gy), with reporting heterogeneity (mean ± SD vs. median [range]). Some cohorts used staged or repeat radiosurgery for larger or residual nidus volumes.

**Table 4:** Summary of studies on stereotactic radiosurgery (SRS) for pediatric brain arteriovenous malformations (bAVMs)

Study	*N*	Radiosurgery	Platform	Margin Dose	Grade (SM)	Nidus	Obliteration (Notes)
Ding [9]	176	88 (50%) alone; 88 micro+radio (50%)	Gamma Knife Radiosurgery	22.3 ± 3.5 Gy (resected) vs. 22.6 ± 2.7 Gy; Max dose ~ 39 Gy	*Low 87 (49.5%)*, *Int 76 (43.2%)*, High 13 (7.4%)	V 2.6 ± 2.0 cm3 (Resect); 2.6 ± 3.3 cm3 (Unresect); D 2.0 ± 0.8 cm	84% Obliteration; resected
Patibandla [10]	28	28 (100%)	Gamma Knife Radiosurgery	19.4 ± 2.3 Gy; Max dose 36.9 ± 6.4 Gy	Low 0 (0%), Int 0 (0%), *High 28 (100%)*	V 5.9 ± 4.4 cm3; D 3.8 ± 0.8 cm	36% Obliteration; 3.2% hemorrhage; 3.5% morbidity/mortality; ruptured higher obliteration
Starke [11]	357	357 (100%)	Gamma Knife Radiosurgery	21.0 ± 3.6 Gy (5–35)	*Low 147 (41.1%)*, *Int 167 (46.8%)*, High 43 (12.1%)	V 3.5 ± 3.3 cm3; D 2.3 ± 1.5 cm	63% Obliteration; 1.4% hemorrhage; 0.8% mortality; ≥22 Gy improved outcomes; no embolization favored success
Umansky [13]	12	12 (100%)	Frame-based LINAC SRS	21.49 Gy (14.39–27.51); Max dose 27.77 Gy (18.93–32.52)	Low 3 (25%), Int 2 (16.7%), *High 7 (58.3%)*	V SRS target 0.6 cm3 (0.1–7.3); V reduction after Onyx 66.7%	71% Obliteration; transient edema only; mRS improved 2→0; no hemorrhage or mortality
Antkowiak [18]	22	3 (13.6%)	Radiosurgery (Not Specified)	16–22 Gy	*Low 15 (68.3%)*, Int 3 (13.6%), High 4 (18.2%)	D 0–3 cm 91%; 3–6 cm 9.1%; >6 cm 0%	2/3 66.7% Obliteration (Endo+Radio); 10/12 83.3% Overall, Micro 5 100%, Endo-only 2 100%, Micro+Endo 1/2 50%
Burke [19]	539	539 (100%): SRS-only *n* = 448, E + SRS *n* = 91	Gamma Knife SRS	20 Gy; Max dose ~ 36–38 Gy	*Low 213 (39.5%)*, *Int 243 (45.1%)*, High 83 (15.4%)	V 5.3 cm3 (SRS-only) vs. 8.4 cm3 (E + SRS)	64.3% Obliteration (Overall); SRS-only (307/448, 68.5%), SRS + E (39/90, 43.3%); embolization reduced cumulative obliteration
Lim [22]	58	10 (17.2%)	Gamma Knife Radiosurgery	NR	*Low 40 (68.9%)*, Int 13 (22.4%), High 5 (8.6%)	D <3 cm 79.3%; 3–6 cm 17.2%; >6 cm 3.4%	81% Obliteration; 13% recurrence; 3% rebleeding; 86% favorable outcome; two deaths
Flores-Milan [26]	27	4 (14.8%): Rupt (3), Unrupt (1)	Stereotatic Radiosurgery (Not Specified)	18 Gy (SRS)	*Low 17 (62.9%)*, Int 6 (22.2%), High 4 (14.8%)	D: Rupt 2.1 cm (IQR 1.5–2.8); Unrupt 2.9 cm (IQR 2.2–3.5)	4/27 14.8% SRS only, 23/27 85.2% Micro only Obliteration
Garcia [27]	83	83 (100%)	Gamma Knife Radiosurgery	19 Gy (first and repeat, isodose line 50%)	*Low 36 (43.3%)*, *Int 40 (48.2%)*, High 7 (8.4%)	V 4.5 cm3 (first SRS), 1.6 cm3 (repeat SRS)	51% Obliteration; 45% favorable; 12% latency hemorrhage; 30% RIC; 7% cyst; 18% retreatment; one death
Kim [28]	95	95 (100%)	CyberKnife (Robotic LINAC SRS)	17.5 Gy (IQR 14.2–20)	Low 17 (18.1%), Int 34 (36.2%), *High 43 (45.7%)*	V 10.0 ± 11.88 cm3 (range 0.11–71.86)	53% Obliteration; 32% ARE; hemorrhage highest in VMS; 1% mortality

Studies in which 100% of patients were treated with radiosurgery are shown in gray and bold. The predominant Spetzler–Martin (SM) grade distribution within each cohort is also highlighted. The radiosurgery percentage is the proportion of patients in each study treated with SRS (either as monotherapy or combined with embolization). Margin dose is reported as mean ± SD, median (range/IQR), or as specified in the original publication. Nidus characteristics include volume (V, cm³) and/or maximum diameter (D, cm). Obliteration refers to angiographic or radiographic AVM cure as defined by each study, with additional notes on hemorrhage, adverse radiation effects (ARE), radiation-induced changes (RIC), recurrence, retreatment, and mortality when reported. Abbreviations: D = Diameter; E = embolization; GKRS = Gamma Knife radiosurgery; IQR = interquartile range; LINAC = linear accelerator; mRS = modified Rankin Scale; NR = not reported; RIC = radiation-induced changes; SFED = single-fraction equivalent dose; SRS = stereotactic radiosurgery; SSF = staged stereotactic fractionation; SSU = single-session uniform; V = Volume; VMS = volume-modulated staged radiosurgery

### Recurrence after apparent cure

Recurrence was examined in seven cohorts **(**Table 5**)** [14, 15, 17, 22–24, 26]. Recurrence after angiographically confirmed microsurgical cure was reported, with rates ranging from 0.9% (Deng [15], hemorrhage-focused surgical cohort, 4.3-year follow-up) to 29.4% (Järvelin [23], pediatric subset only, 5.7-year follow-up), and 10.4%–16.7% in the largest dedicated recurrence series (Copelan [17]). Across all seven cohorts, the overall recurrence rate ranged from < 1% to 29%. Younger age at treatment and hemorrhagic presentation were consistently associated with recurrence. Long-term surveillance cohorts demonstrated that recurrence may occur many years after apparent cure (Oulasvirta 2023 [24]: events detected 14–26 years post-treatment; Järvelin [23]: events up to 30 years). Recurrence after SRS has been reported in only one of the seven cohorts: Lim et al. [22] reported 2/12 (16.7%) recurrences in the SRS subgroup at 5.3-year follow-up within a multimodality series. No dedicated SRS-alone pediatric recurrence series was identified; accordingly, post-SRS recurrence rates cannot be independently quantified from the available data.

**Table 5:** Studies reporting recurrence in pediatric brain arteriovenous malformations (bAVMs)

Study	Treatment	Hemorrhage, 1st presentation	Recurrence
Al-Smadi [14]	Micro+Endo 73.5%, Micro only 26.5%	20 (59%)	1/34 (2.9%), after 1y post-Micro
Deng [15]	Micro 111 (100%)	111 (100%)	1/111 (0.9%), at 9mo post-Micro
Copelan [17]	Micro 115(100%) ± pre-Endo/SRS	72 (63%)	12/115 (10.4%, Overall), 12/72 (16.7%, associated with Hemorrhagic), 21.4% 5 years FU
Lim [22]	Micro 36 (62.1%), SRS 17.2%, Endo 3.4%, Surveillance 17.2%	51 (87.9%)	6/46 (13%, Overall), 4/35 (11.4%, Micro), 2/12 (16.7%, SRS), 5.3 years FU
Järvelin [23]	Micro 135 (100%), preoperative Endo 35.3%	79 (58.5%)	5/17 (29.4%), 5.7 years FU (associated with Hemorrhagic)
Oulasvirta [24]	Micro 37 (90.2%), combined: 14 (34.1%)	29 (70.7%)	2/41 (4.9%), 3/42 (7.1%, 1 Osler-Weber-Rendu), 26 years FU
Flores-Milan [26]	Micro 23 (85.2%), pre-Endo 47.6% (ruptured group)	21 (77.8%)	2/27 (7.4%, Overall), 2/21 (9.5%, Hemorrhagic cohort), 3 and 5 years FU

Recurrence rates are presented as number/total (%), with time to recurrence or follow-up duration when available. In the included studies, recurrences were reported predominantly after microsurgical resection, often in patients with prior hemorrhagic presentation. Abbreviations: Micro microsurgery; Endo endovascular embolization; SRS stereotactic radiosurgery; FU follow-up; y year(s); mo month(s); pre-Endo preoperative embolization

## Discussion

This systematic review synthesizes contemporary retrospective evidence on pediatric bAVM management and underlies *three principal observations*. *First*, across published cohorts over the past decade, hemorrhage remains the dominant clinical presentation, particularly in surgery-dominant and recurrence-focused microsurgical series, underscoring the clinical relevance of referral and treatment selection driven by rupture in pediatric practice. *Second*, the aggregate angioarchitectural profile of treated pediatric bAVMs is skewed toward low- and intermediate-grade lesions, with Spetzler–Martin (SM) I–II and III comprising the majority of reported cases (44.1% and 38.4%, respectively), whereas SM IV–V lesions constitute a smaller but clinically consequential subgroup (17.5%) that is disproportionately represented in conservative cohorts. *Third*, although most retrospective series report high obliteration rates in their selected populations, the magnitude of reported “success” varies systematically by modality, case mix, and outcome definition, limiting cross-modality inference and reinforcing the need for standardized reporting and long-term surveillance in children.

### Treatment effects, patient selection, and outcomes

Across modalities, the observed gradients in obliteration and morbidity largely reflect selection practices rather than directly comparable treatment effects. Microsurgical series—typically enriched for low- and intermediate-grade ruptured lesions—report high immediate angiographic cure rates, including cohorts with near-universal obliteration when postoperative DSA is used for confirmation. In contrast, radiosurgery cohorts, which include a broader grade distribution and routinely contend with latency to obliteration, report obliteration rates of approximately 51%–68.5% across the three largest unselected pediatric SRS series (Starke [11], Burke [19], Garcia [27]), with markedly lower rates (43.3%) when SRS follows prior embolization (Burke [19]). Latency-period hemorrhage rates of approximately 1.1%–3.2% per series were consistently reported. These differences are expected given inherent modality characteristics and referral patterns; however, retrospective studies frequently conflate treatment strategy with baseline risk because cohorts differ in SM grade distribution, deep/eloquent location, nidus size reporting, and rupture composition. This reinforces the interpretive boundary that the existing evidence base is informative for real-world outcomes within modality-specific practice contexts, but it is not designed to support causal superiority claims among microsurgery, embolization, and radiosurgery.

### Radiosurgery: efficacy and hemorrhage latency 

The radiosurgery literature consistently supports SRS as a durable option for selected pediatric bAVMs, particularly for lesions not optimally suited for resection. However, the results identified *two practical considerations. First*, hemorrhage latency is not negligible across cohorts and must be explicitly addressed in counseling and shared decision-making. *Second*, sequencing with embolization appears to influence radiosurgical outcomes in multiple datasets; international registry data summarized here show lower obliteration when SRS follows prior embolization, a pattern that is clinically plausible given potential nidus fragmentation and altered target definition. While these observations do not prove harm from embolization, they support more granular reporting of pre-SRS embolization intent (adjunctive vs. curative), extent, and angiographic target evolution—variables that are inconsistently captured across retrospective series.

### Embolization and combined management

Endovascular series in pediatric bAVMs span fundamentally different objectives—curative embolization in carefully selected low-grade lesions versus adjunctive embolization to facilitate surgery or radiosurgery—yet they are often reported under a single “embolization” label. This heterogeneity complicates the synthesis of obliteration and complication rates and likely contributes to the wide range of reported outcomes across studies. Similarly, multimodality cohorts vary in whether treatment is planned, staged, or salvage-driven. The absence of triple-modality reporting in the classifiable cohort **(**Fig. 4**)** may reflect reporting conventions rather than a true clinical absence, and basing treatment categorization on retrospective publications can obscure real-world sequencing and decision points.

### Pediatric-specific recurrence rates and long-term surveillance 

A central pediatric implication from this review is the nontrivial recurrence signal after “cure,” particularly after microsurgical resection with angiographic confirmation. Among cohorts that explicitly tracked recurrence, overall rates ranged from 0.9% (Deng [15], 4.3-year follow-up) to 29.4% (Järvelin [23], pediatric subset, 5.7-year follow-up). The largest dedicated recurrence series (Copelan [17]) reported recurrence rates of 10.4%–16.7%, depending on hemorrhagic status. Pediatric subsets within mixed-age cohorts consistently showed substantially higher recurrence rates than those in adult populations. Importantly, long-term follow-up series document recurrences many years after the detection of apparent cure events: 14–26 years post-treatment in Oulasvirta (2023) [24] and up to 30 years in Järvelin [23], with all recurrences ultimately successfully re-treated. Post-SRS recurrence data are sparse: among the seven recurrence-reporting cohorts, only Lim et al. [22] reported a distinct SRS subgroup recurrence rate (2/12, 16.7%, 5.3-year follow-up), and no dedicated SRS-only pediatric recurrence series was identified. These observations support two practice-facing points aligned with the literature: [1] “cure” in pediatric bAVM is time-dependent, and [2] surveillance strategies should be explicit, durable, and preferably standardized, with careful attention to imaging modality and interval. The current literature also suggests that recurrence risk is not uniform [6, 7]; younger age and hemorrhagic presentation are repeatedly observed among recurrences, arguing for risk-stratified follow-up intensity in children.

### Limitations

The findings of this systematic review are constrained by the same methodological limitations that characterize the underlying literature. All included studies were retrospective, with inherent selection bias and confounding by indication. Outcome definitions—particularly obliteration—were inconsistent (DSA-confirmed cure vs. MRI-based assessments), and follow-up ascertainment varied across cohorts, limiting comparability and precluding robust pooled estimates. Notably, no minimum follow-up duration was pre-specified for inclusion because the criterion required only that at least one post-management outcome be reported. This permissive threshold introduces substantial heterogeneity in follow-up duration (ranging from 3 months to a median of 19.1 years across cohorts) and limits the comparability of recurrence and obliteration estimates across studies. Angioarchitectural reporting beyond SM grade was incomplete and nonuniform, with nidus size reported as diameter or volume using heterogeneous thresholds. Finally, reporting of functional outcomes and complications was variably structured and often not standardized across modalities. These limitations substantiate the rationale for a descriptive synthesis and reinforce that inference should remain modality- and cohort-contextual rather than comparative.

### Clinical implications and future research 

Despite these limitations, the retrospective evidence provides actionable signals for clinical counseling. Microsurgery in appropriately selected pediatric bAVMs yields high cure rates but is not without morbidity; radiosurgery offers meaningful long-term obliteration with an expected latency interval and attendant hemorrhage risk; and embolization outcomes depend strongly on whether the goal is cure or adjunctive therapy. The recurrence data provide the most pediatric-specific management implication: long-term post-cure surveillance should be treated as a core component of management rather than an optional adjunct. Moving forward, the field would benefit most from harmonized reporting standards for pediatric bAVM cohorts: minimum datasets for angioarchitectural variables, explicit treatment sequencing and intent, standardized definitions of obliteration (with a clear imaging modality), and time-to-event reporting for hemorrhage, obliteration, and recurrence. This would substantially improve interpretability and enable more defensible comparative effectiveness analyses, even within observational designs.

## Conclusion

In this systematic review of contemporary retrospective cohorts, pediatric bAVM management remains largely guided by hemorrhagic presentation and lesion architecture, with most treated cases classified as Spetzler–Martin grades I–III and a smaller but clinically important fraction of high-grade lesions concentrated in conservative cohorts. Across selected populations, microsurgical series generally report high immediate cure rates, whereas radiosurgery provides meaningful long-term obliteration but carries a nontrivial risk of hemorrhage during the latency period and variable outcomes influenced by prior embolization and lesion characteristics; endovascular results remain highly dependent on whether embolization is pursued with curative intent or as an adjunct. A key pediatric-specific finding is that recurrence after apparent cure is not rare and may occur years after angiographic obliteration, supporting the need for explicit, long-term, and risk-stratified surveillance protocols. Collectively, these data inform counseling by framing modality-specific outcomes within their selection context and reinforce the necessity of standardized definitions, harmonized reporting, and time-to-event analyses to enable more defensible comparative effectiveness inference in future pediatric bAVM studies.

## Data Availability

No datasets were generated or analysed during the current study.
